# An approach to exploring patterns of imbalance and potential missingness in reports of the randomized baseline values for primary outcomes measurable at baseline in randomized controlled trials for meta-analyses

**DOI:** 10.1186/s12874-022-01620-x

**Published:** 2022-05-28

**Authors:** Eun-Gee Park, Seokyung Hahn

**Affiliations:** 1 Interdisciplinary Program in Medical Informatics, Seoul National University College of Medicine, Seoul, South Korea; 2 Department of Human Systems Medicine, Seoul National University College of Medicine, Seoul, South Korea; 3 Division of Medical Statistics, Medical Research Collaborating Center, Seoul National University Hospital, Seoul, South Korea; 4 Institute of Health Policy and Management, Medical Research Center, Seoul National University, Seoul, South Korea

**Keywords:** Meta-analysis, Randomized controlled trials, Baseline imbalance, Baseline heterogeneity, Primary outcome

## Abstract

**Background:**

This study evaluated the adequacy of randomization in randomized controlled trials by investigating baseline differences in the primary outcome when a meta-analysis employed an outcome whose baseline level was measurable.

**Methods:**

We retrieved Cochrane reviews published during one year. We calculated the proportion of studies that reported randomized baseline values for the primary outcome. The standardized mean difference (SMD) was used to assess baseline imbalance and heterogeneity. We explored ranking-ordered forest plots using a normal cumulative probability curve as a guideline representing well-performed randomized trials. When skewness was suggested, a funnel plot was drawn to assess whether there was a significant linear trend.

**Results:**

In 10 of 18 meta-analyses, more than 25% of trials did not report randomized baseline values of the primary outcomes. Three meta-analyses indicated baseline imbalance (*P* < 0.1) and three showed substantial heterogeneity (*I*^2^ > 60%). Four meta-analyses with forest plots suggesting a skewed SMD distribution also showed a linear trend on their standard errors on the funnel plot.

**Conclusions:**

If the primary outcome is measured at baseline, it is essential to explore the full scope of baseline imbalance among the trials. This could help understand patterns of bias, including missingness, for designing adjustment.

**Supplementary Information:**

The online version contains supplementary material available at 10.1186/s12874-022-01620-x.

## Background

Randomized controlled trials (RCTs) use random allocation to minimize the baseline difference between arms. Randomization ensures the comparability of each arm by balancing unmeasured prognostic factors and confounding variables. Therefore, when randomization is properly conducted, differences in baseline characteristics between arms should occur only by chance. If there exist chance imbalances, we should adjust for the baseline covariates by using a statistical approach such as analysis of covariance (ANCOVA) [1]. The Consolidated Standards of Reporting Trials (CONSORT) guidelines require RCTs to report baseline demographic and clinical characteristics in each arm [2].

Under the condition that comparison groups are well balanced after random assignment in RCTs, systematic reviews and meta-analyses can infer unbiased pooled estimates. According to the Preferred Reporting Items of Systematic reviews and Meta-Analyses (PRISMA) guidelines [3, 4], it is a norm that the risk of bias [5] of RCTs is assessed as part of the systematic review process, which primarily includes an evaluation of the methods used for random sequence generation and allocation concealment. The recently revised Cochrane tool for assessing the risk of bias in randomized trials (RoB 2.0) recommends assessing baseline differences between intervention groups along with the potential for bias arising from the randomization process [6, 7].

When we conduct a meta-analysis of RCTs, although we expect a good balance in values of the primary outcomes to be achieved after randomization between intervention groups, it is not always very plausible that this condition holds true. When evaluating an outcome that is an event such as mortality, there is only the ‘endpoint’ to account for, and no baseline status of the outcome itself can be measured. In such a case, it may be especially important to assess the baseline status of important covariates among the randomized patients in a meta-analysis [8]. If we evaluate an outcome whose baseline values are measurable and of concern, such as pain or symptom scores that are measured on a continuous scale, it is essential to assess the primary outcome’s baseline balance between groups. Thus, it is also critical for the primary RCT authors to report these values so that they can be evaluated by the meta-analyst and be taken into account if any considerable imbalance is noted.

As an example of the importance of this issue, a meta-analysis of the effects of calcium supplementation on body weight showed that the baseline body weight was significantly lower in the treatment group than in the control group [9]. The authors proposed using an ANCOVA-type meta-regression method to adjust for the baseline imbalance and demonstrated that the apparent treatment effect was, in fact, due to the baseline difference [10]. Another meta-analysis of pain after epidural steroid injections in low back pain patients reported a significantly lower mean baseline pain score in the intervention group [11]. The authors also adjusted for the baseline pain scores and emphasized the need to adjust for baseline imbalances, which might change the conclusion of meta-analyses. The presence of a systematic imbalance in baseline values after randomization poses questions regarding how well the randomization process in clinical trials functions in reality and whether imbalanced allocation may be related to specific factors, such as the type of intervention or outcome.

In addition to the overall imbalance of baseline values, the extent of heterogeneity has also been suggested as a sign of bias against randomization [12]. A Cochrane review of spinal manipulative therapy for chronic low back pain showed that there was no significant pooled difference between groups, but significant heterogeneity was present in baseline back pain scores, as some trials with a baseline imbalance between groups were included in the meta-analysis [8].

In this study, we first attempted to investigate the proportion of trials that reported the randomized baseline values for the primary outcome, when their meta-analysis employed an outcome for which the baseline level was measurable, together with the characteristics of the meta-analysis. The balance between groups after random assignment was examined for the reported values. Finally, we aimed to devise an exploratory approach based on graphical presentations to explore the potential for selection bias in the RCTs included in a meta-analysis.

## Methods

### Study selection

We retrieved Cochrane reviews published in 2018 from the Cochrane Library. The inclusion criteria were systematic reviews that contained a meta-analysis of more than 10 RCTs and clearly defined the primary outcome. We selected meta-analyses that used primary outcomes of quantitative measures, the baseline level of which should be assessed. One meta-analysis with the largest number of RCTs per systematic review was chosen if multiple meta-analyses of primary outcomes were eligible within a study.

### Data extraction

One author extracted data and the second author cross-checked the data, and a discussion was held to resolve any questions by consensus. For each meta-analysis, we extracted the definitions of PICO (patients, intervention, comparator, and the primary outcome), the type of effect measures, and the number of RCTs included. For each trial, we extracted the number of participants in each arm and the mean and standard deviation (SD) of the baseline values for the primary outcome. If the mean and SD were not reported, we collected other forms of summary statistics, such as the median and interquartile range.

### Statistical analysis

We first identified RCTs that reported baseline values for the primary outcomes and confirmed that the reported values could be considered as results from a randomized sample. For studies that reported baseline values only for the analyzed patients (not for all the randomized patients), we considered the results as arising from a randomized patient sample if the dropout rate was less than 10%.

We calculated the baseline difference for each trial using the standardized mean difference (SMD) to evaluate the balance on a common scale across meta-analyses considering different outcomes with different ranges of magnitude. Baseline values reported as the median with interquartile range were not included in the calculation of SMD measures. The baseline mean differences were coded so that an SMD < 0 indicated that the patients in the intervention group of interest were in a better condition at baseline (e.g., having lower symptom scores or higher activity scores). When it was difficult to judge which direction would indicate a better condition based on the outcome alone, the characteristics of the disease and the intervention of interest were considered in the context of the study objective; for example, a lower preoperative glucose level implies a better condition when investigating the perioperative hyperglycemic response to surgical stress.

We calculated the pooled SMD, using the weight of inverse variance of the baseline mean difference from each trial, with the 95% confidence interval (CI). Statistical heterogeneity across trials within a meta-analysis was evaluated using the *I*^2^ statistic [13]. We regarded *I*^2^ values of 0–40%, 30–60%, 50–90%, and 75–100% as indicating insignificant, moderate, substantial, and considerable heterogeneity, respectively [14]. We also examined the proportion of trials with negative SMD values in each meta-analysis.

For each meta-analysis, the SMD and its 95% CI for the included trials were presented in descending order in a forest plot. A cumulative normal distribution function was overlaid, assuming that the distribution would have a mean of zero and the same variability as that of the pooled SMD, representing the curve that one would expect with only sampling error when the observed baseline differences were obtained from well-performed randomized trials. We visually explored the location and the shape of the observed values using the cumulative probability curve as a guideline. Symmetry was confirmed first with the location of the median value being zero, and a pattern of deviation of interval estimates for the SMD from the curve was identified. We considered there to be a sign of skewness when the location of the median value disaccorded with zero and the trial estimates deviated from the imposed guideline in one direction. When some skewness was suggested, we further drew funnel plots of SMDs with their standard errors (SEs). We assessed whether there was any notable trend in SMDs [15].

Statistical analyses were performed using R version 3.6.1 (R Foundation for Statistical Computing, Vienna, Austria).

## Results

### Study characteristics of meta-analyses

**Fig. 1:**
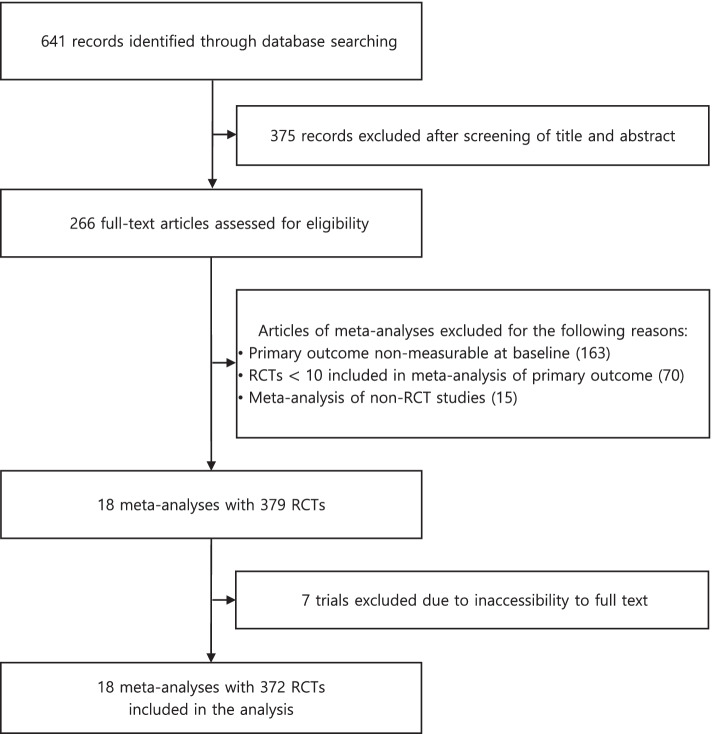
Flow diagram of the selection process. RCT, randomized controlled trial

The flow diagram of the study selection process is reported in Fig. 1. From 641 Cochrane reviews, 18 meta-analyses on 372 trials met our selection criteria. The characteristics of each meta-analysis are summarized in Table 1. Fifteen meta-analyses used subjective primary outcomes such as pain or symptom scores (#3-#7, #9-#18). Five of the eight meta-analyses using active controls mainly targeted patients receiving surgical care (#1, #2, #16-#18). Eight of the other 10 meta-analyses using placebo or standard of care were studies of patients receiving medical care (#3, #4, #6-#8, #11-#13). Nine of the 13 meta-analyses on pharmacological interventions used mean differences (#1, #2, #4, #7, #10, #14, #16-#18), and all five meta-analyses on non-pharmacological interventions used SMDs (#3, #5, #9, #11, #13). Those five studies evaluated their outcome measures primarily at the end of follow-up. Five of the other six studies with evaluations at the end of follow-up used pain as the primary outcome (#14-#18).

**Table 1:** Characteristics of the meta-analyses included in this study

Meta-analysis	Patients	Intervention	Comparator	Primary outcome	Effect measure	Follow-up evaluation
# 1 [16]	Patients of all ages with periodontal infrabony defects	Autologous platelet concentrates	Active control	Probing depth	MD	Change in score
# 2 [17]	Adults undergoing surgery	Dexamethasone	Active control (6), placebo (2)	Glycemic measurement	MD	Change in score
# 3 [18]	Adults exposed to traumatic events	Psychological therapy	No treatment, standard of care	Post-traumatic stress disorder	SMD	Final value
# 4 [19]	Patients 0–55 years of age with eczema	Probiotics	Placebo	Symptoms of eczema	MD	Change in score
# 5 [20]	Adults (over 18 years) with stroke	Electromechanical and robotic-assisted training	Active control	Activities of daily living	SMD	Final value
# 6 [21]	Adults with functional dyspepsia	Prokinetic	Placebo	Symptom scores	SMD	Change in score
# 7 [22]	Male patients with lower urinary tract symptoms	Phosphodiesterase inhibitors	Placebo	International Prostate Symptom Score	MD	Change in score
# 8 [23]	Adults and children receiving peritoneal dialysis	Low glucose degradation product dialysate	Standard of care	Residual renal function	SMD	Final value
# 9 [24]	Adults (over 18 years) with stroke	Mirror therapy	Active control	Motor function	SMD	Final value
# 10 [25]	Male patients with lower urinary tract symptoms	Naftopidil	Active control	International Prostate Symptom Score	MD	Final value (3), change in score (5)
# 11 [26]	Older adults with dementia	Music-based therapeutic interventions	Standard of care (7), active control (2)	Behavior problems	SMD	Final value
# 12 [27]	Adults with attention deficit hyperactivity disorder (ADHD)	Amphetamines	Placebo	ADHD symptom severity: clinician-rated	SMD	Final value (2), change in score (6)
# 13 [28]	Children aged 2–19 undergoing any needle-related medical procedure	Distraction	Standard of care	Self-reported pain	SMD	Final value
# 14 [29]	Adults undergoing any type of surgery	Intravenous ketamine	Placebo	Pain intensity at rest	MD	Final value
# 15 [30]	Surgical procedure under general anesthesia in adults	Intravenous lidocaine	Placebo, no treatment	Pain score at rest	SMD	Final value
# 16 [31]	Adults undergoing intra-abdominal surgery	Epidural analgesia	Active control	Pain score at rest	MD	Final value
# 17 [32]	Adults or adolescents with suspected appendicitis	Laparoscopic appendectomy	Active control	Pain intensity	MD	Final value
# 18 [33]	Adults undergoing dental surgery	pre-emptive opioids	Active control	Early acute postoperative pain	MD	Final value

*MD* mean difference, *SMD* standardized mean difference

### Reporting of baseline values for the primary outcome

**Fig. 2:**
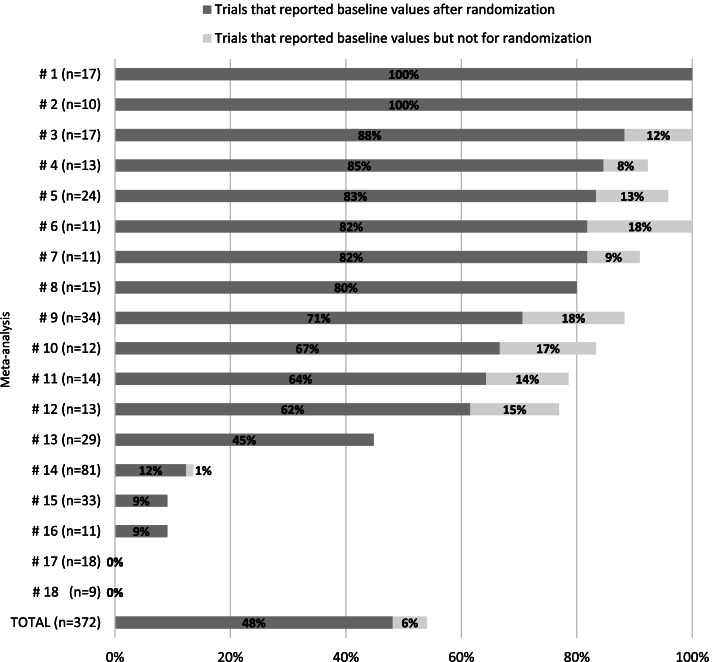
The proportion of RCTs that reported baseline values for the primary outcome in each meta-analysis

Baseline values for the primary outcome were not reported in 171 of the 372 trials (46%). Among 201 trials that reported baseline values, 22 only reported the baseline values for analyzed patients with a dropout rate greater than 10%. Therefore, we identified 179 trials (48%) that reported baseline values for the randomized patients. Six out of the 179 trials reported baseline values as the median with interquartile range. In eight of the 18 meta-analyses, fewer than 20% of trials did not report randomized baseline values, whereas in two studies, 100% of trials reported the values. In five other meta-analyses, the baseline values were not reported in 29–55% of trials. Only a small proportion (0–12%) of trials reported baseline values in the other five meta-analyses (#14-#18) that assessed postoperative pain in surgical patients, for whom the baseline pain score before surgery was not of concern (Fig. 2). The 10 meta-analyses (#9-#18), in which relatively few trials reported baseline values, evaluated the outcome measures mainly based on follow-up scores.

### Baseline imbalance and heterogeneity

**Fig. 3:**
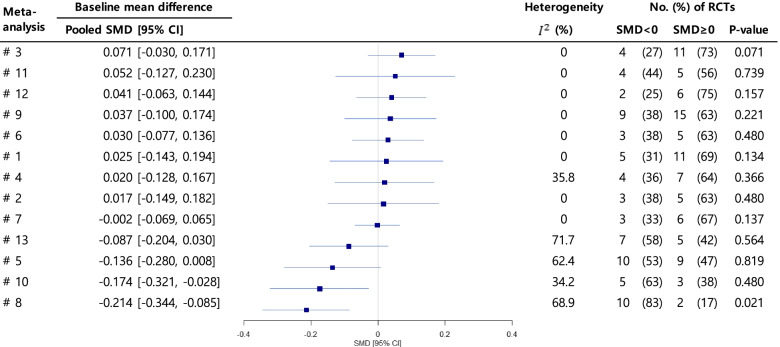
Results of baseline differences after randomization for the primary outcome in each meta-analysis. The median baseline values obtained from 6 trials were not included in the analysis. *P*-value was produced by one-proportion z-test. RCT, randomized controlled trial; SMD, standardized mean difference; CI, confidence interval

In terms of the weighted pooled SMD, two meta-analyses indicated significant baseline mean differences, with a *p*-value < 0.05 (#8: SMD = − 0.214 [95% CI = − 0.344, − 0.085]; #10: SMD = − 0.174 [95% CI = − 0.321, − 0.028]). Another meta-analysis showed significance with a *p*-value < 0.1 (#5: SMD = − 0.136 [95% CI = − 0.280, 0.008]), and two meta-analyses showed *p*-values between 0.1 and 0.2 (#3: SMD = 0.071 [95% CI = − 0.030, 0.171]; #13: SMD = − 0.087 [95% CI = − 0.204, 0.030]). There was substantial heterogeneity in the observed baseline differences of primary outcomes across the trials in three meta-analyses (#5: *I*^2^ = 62.4%; #8: *I*^2^ = 68.9%; #13: *I*^2^ = 71.7%), and moderate heterogeneity was found in two meta-analyses (#4: *I*^2^ = 35.8%; #10: *I*^2^ = 34.2%) (Fig. 3). The four meta-analyses (#5, #8, #10, #13) that showed both imbalance and heterogeneity in the baseline values assessed their outcomes primarily based on follow-up scores. In 10 of the 13 meta-analyses reported in Fig. 3, a large proportion of trials (63–83%) showed baseline imbalance either in a negative or a positive direction. Two of them showed significant proportional differences, at the 0.05 level (#8) and at the 0.1 level (#3). Another three showed *p*-values between 0.1 and 0.2 (#1, #7, #12).

### Graphical representations of baseline differences

**Fig. 4:**
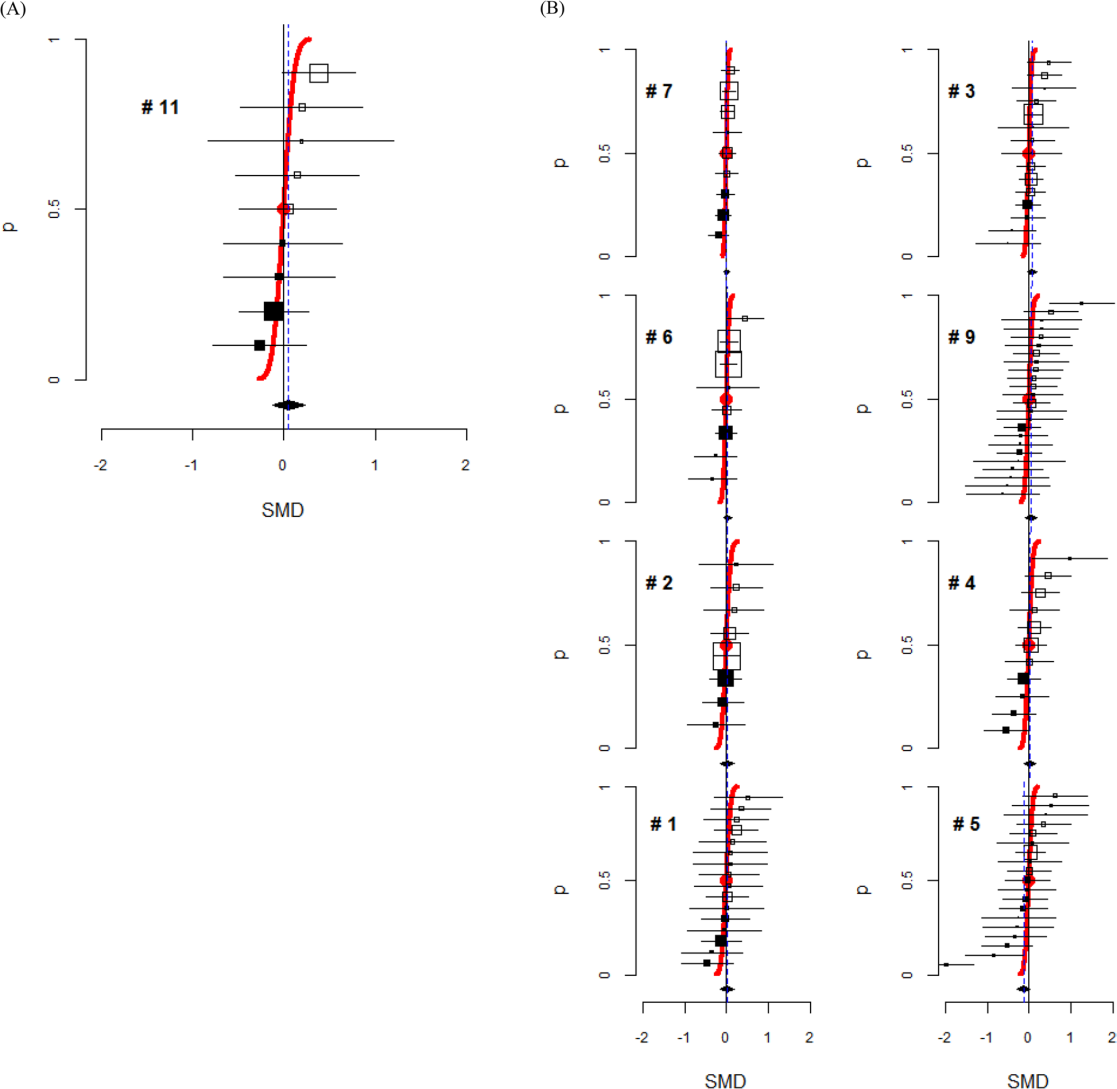
Forest plots for a meta-analysis without skewness of the distribution. **A** Standard forest plot for baseline mean differences. **B** Forest plots for other studies. The vertical axis represents a probability from 0 to 1. The horizontal axis represents a baseline standardized mean difference (SMD), and a negative SMD value indicates that the treatment group is better at baseline. The size of the square shows the weight of trials. A black square represents a negative direction, and a white square represents a positive direction. The polygon indicates a pooled estimate $(\widehat b)$. The red line follows the cumulative normal distribution function with a mean of zero and variance $\mathit{\operatorname{var}}(\hat{b})$. The red circle indicates the median and mean value of the distribution, which is SMD = 0

**Fig. 5:**
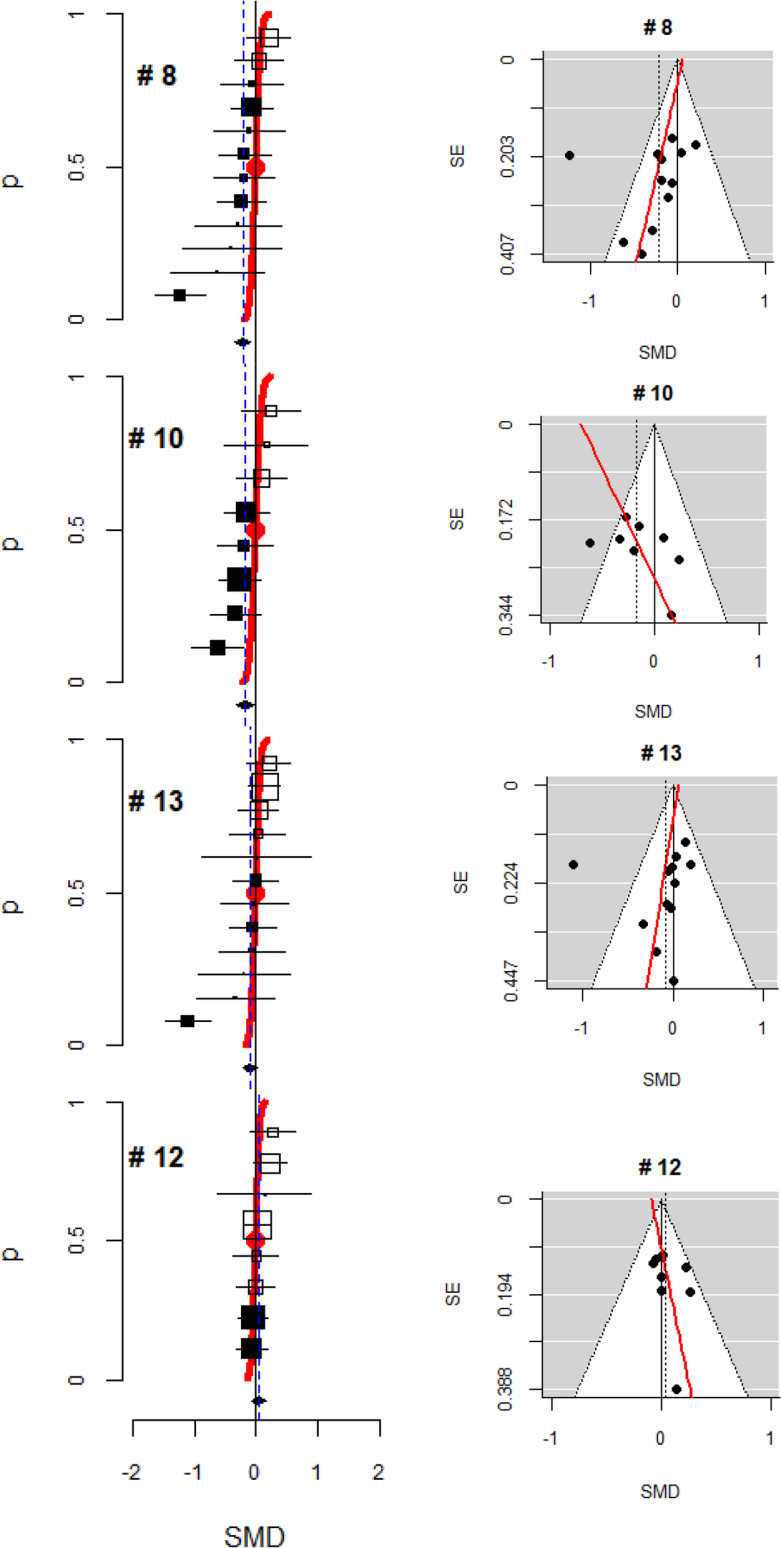
Forest plots and funnel plots for a meta-analysis with skewness of the distribution. In the funnel plot, the x-axis is the standardized mean difference (SMD) at baseline and the y-axis is the standard error (SE). The pseudo 95% confidence interval line around the SMD equaling zero and the pooled SMD are presented

Figure 4A presents a forest plot for randomized baseline mean differences, where the median value was observed to be zero and the interval estimates for the SMD were in line with the cumulative normal distribution function. In five meta-analyses (#7, #6, #2, #1, #3), while the interval estimates followed the cumulative probability curve closely, the median value disaccorded with zero. In three other meta-analyses (#9, #4, #5), the deviation of the SMD estimates from the curve was wider, indicating greater variance than what was assumed, and some intervals were found to be out of line (Fig. 4B). Some skewness was suggested in the distribution of the baseline SMDs for the other four meta-analyses; three were skewed to the right (#8, #10, #13) and one to the left (#12). The funnel plots for those four meta-analyses exhibited asymmetry, with a linear trend of SMDs according to their SEs; larger differences in SMDs were found as the SEs became greater in three studies (#8, #13, #12), whereas in one study, larger differences in SMDs tended to be found when the SEs were smaller (#10) (Fig. 5).

## Discussion

Our finding suggests that randomized baseline values for the primary outcome were not reported in about half of trials (52%). The majority of them (72%) were studies of postoperative pain management in surgical patients, for whom baseline pain scores would be irrelevant. Therefore, we excluded those trials from our evaluation of the randomized baseline status of the primary outcome. After excluding these trials, the actual proportion of trials that did not report randomized baseline values for the primary outcome was 25%. One other meta-analysis on the effectiveness of distracting children during needle-related medical procedures reported baseline pain scores in 45% of trials, although the primary outcome’s baseline value may also have been irrelevant. In this case, the reported baseline pain might be considered as an important prognostic factor that could influence the outcome, rather than a baseline value of the primary outcome itself, but 55% of trials still did not report values for this variable. A prior study of baseline variables that are predictive of outcomes in a meta-analysis found that 40% of trials did not report some of the baseline demographic data [34].

All the studies with significant baseline imbalances (3/13) or heterogeneity (3/13) were found to use a final value measured at the end of follow-up instead of changes in scores from baseline. We also found that all meta-analyses with a large proportion of missing baseline values (more than 30%) evaluated their outcome measures at the end of follow-up, which would be natural considering the unavailability of baseline values. If the baseline scores for the randomized patients are unbalanced in a clinical trial, ANCOVA is the optimal statistical method for adjustment [35]. When conducting a meta-analysis, we may also need to adjust for baseline differences when a significant imbalance occurs [10, 36]. However, when the randomized baseline values for the outcome are not reported in trials, and thus are not available for such an analysis, any existing method of statistical analysis would not be practical to apply.

In the forest plot, the SMDs of baseline outcome values for trials were positioned in descending order according to the value of k/(n + 1), where k is the rank of the trial given a total of n trials. In this way, each observed SMD could be considered as the crude k/(n + 1) × 100th percentile in the forest plot as an empirical version of a cumulative probability plot. The overlaid cumulative normal distribution curve can be interpreted as a guideline that would theoretically appear if the true mean baseline difference is zero. The interval estimates of baseline differences for the included trials are, therefore, expected to be approximately in line with the guideline if they were obtained from well-performed randomized trials. In this context, we aimed to identify how the observed baseline differences after randomization deviated from what would be obtained in the ideal situation where randomization was well-performed. In the ideal world, the true mean baseline difference should be fixed as a value of zero for all trials if the randomization was conducted well, with a certain extent of variation that we obtained from a fixed-effect model. A notable observed departure from the guideline curve might then be interpreted as a phenomenon caused by heterogeneity, possibly resulting from variation in the quality of randomization from study to study, or potential missingness in reports of baseline values, as suggested by the presence of a certain pattern, which may also be related to systematic bias in randomization.

Detecting a deviation in the forest plot from the guideline suggests a possibility of selection bias in randomization. Evaluating the pattern of deviation can help us to understand the characteristics of the bias. We found skewness of baseline differences in four meta-analyses; three were skewed to the right and one to the left. The direction of skewness primarily suggests the direction of selection bias that should be examined through characteristics of the disease and the intervention in the context of the study objective. A skewed distribution of baseline differences may additionally suggest a pattern of missingness in the values that could be identified through further investigation of its funnel plot. In those four meta-analyses, a large proportion of included trials did not report the randomized baseline values (20–55%), and therefore, the observed trend may be associated with the missingness. Three of those four meta-analyses showed a trend for the SMDs to increase as the SEs became greater, implying that a majority of the unreported values would likely be large SMD values missing in the opposite direction. Therefore, if the baseline values were fully reported, their observed distribution would more likely have a mean value closer to zero. In contrast, one meta-analysis showed a different pattern, suggesting that the missing values would be found in the same direction where the majority of observed values lay. This means that the distribution of baseline differences after randomization would still have a shifted mean from zero even if the missing values were filled, implying that some systematic subversion in randomization might have occurred in those trials.

We limited our study sample to Cochrane reviews published in 1 specific year. Our study had some limitations in interpreting the randomized baseline differences in the primary outcome and their reporting status in relation to study characteristics. For example, most of the pain outcomes included in our study did not have measurements of baseline status (postoperative pain management in surgical patients) and there was no study on controlling pain by a procedure in patients with pain in our study set, which would have been of interest since baseline pain measurements would be relevant in such a study. Therefore, future research with a broader range of characteristics, involving not only Cochrane reviews but also studies published in other major medical journals, will enable further elaboration of our results.

Our investigation was carried out using SMD measurements to compare the resulting balance in baseline values of the primary outcomes after randomization on a common scale across meta-analyses, the primary outcomes of which could have different magnitudes by their nature. However, if any future meta-analysts wish to use our approach for meta-analyses where the MD would be a more appropriate measure, there would be no need to choose the SMD over the MD unless they intend to compare their results to those of other studies that used a different outcome.

Our suggested approach is a visual exploratory analysis by nature. Since we did not apply any formal test, there might be some concerns about the subjectivity of interpretation, particularly in the determination of skewness. Although limited statistical power would still be a problem, it would be useful to devise a formal meta-analytic test method for skewness that could appropriately be considered in the given context. Furthermore, additional research on how the baseline imbalanced and missed baseline values affect pooled results in meta-analyses should be conducted in more detail as future work.

## Conclusions

In our study, significant baseline imbalances or substantial baseline heterogeneity for the primary outcome commonly occurred after randomization. The Cochrane RoB 2.0 tool [7] recommends assessing imbalance in major key prognostic factors or baseline measures of outcome variables that are unlikely to occur by chance alone. We suggest that if a meta-analysis employs a primary outcome with a measurable baseline level, it is particularly essential to explore the full scope of its baseline imbalance among the trials by using graphical methods that could provide a sense of the likelihood of systematic selection bias and potential patterns of missing reports. These methods could help in determining whether and/or how to effectively adjust for the bias. If the primary studies do not report the relevant data, using any modeling technique for adjustment is of very limited applicability. It is particularly important to make sure that the included trials report those data.

## Supplementary Information

**Additional file 1: Appendix 1.** R scripts for generating graphical representations of baseline differences.

## Data Availability

The data that support the findings of this study are secondary data drawn from already published literature as referenced within the article. The R scripts for the proposed graphical approach are provided in Appendix 1.

## Abbreviations

RCT: Randomized controlled trial
ANCOVA: Analysis of covariance
CONSORT: Consolidated Standards of Reporting Trials
PRISMA: Preferred Reporting Items of Systematic reviews and Meta-Analyses
ROB: Risk of bias
SD: Standard deviation
SMD: Standardized mean difference
CI: Confidence interval
SE: Standard error
