# Supplementary material for: An approach to exploring patterns of imbalance and potential missingness in reports of the randomized baseline values for primary outcomes measurable at baseline in randomized controlled trials for meta-analyses
Source: BMC Med Res Methodol. 2022 May 28;22:154. doi: 10.1186/s12874-022-01620-x (PMC9148458; doi:10.1186/s12874-022-01620-x)
Supplement: Supplementary file 1 — Additional file 1: Appendix 1. R scripts for generating graphical representations of baseline differences. [file 12874_2022_1620_MOESM1_ESM.pdf]

Appendix 1. R scripts for generating graphical representations of baseline differences

```
library(metafor); library(meta)

rcts<-read.csv ("baseline data.csv")

#Contents of variables for the first 6 trials in the dataset#
head(rcts[,c("study", "trt_m", "trt_sd", "nt", "con_m", "con_sd", "nc")])
#    study   trt_m   trt_sd     nt   con_m   con_sd     nc
# 1    1     7.23    0.790000   24   7.25    0.770000   24
# 2    1     7.13    0.880000   30   7.12    0.780000   30
# 3    1     6.75    0.850000   20   7.12    0.720000   20
# 4    1     6.30    2.132875   10   6.40    2.065527   10
# 5    1     7.30    1.370000   13   7.00    1.080000   13
# 6    1     7.28    0.530000   12   7.19    0.710000   12

#study: study number for meta-analysis the trial included to
#trt_m: mean baseline value from treatment group
#trt_sd: standard deviation of baseline value for treatment group
#nt: number of participants included in the treatment group
#con_m: mean baseline value from control group
#con_sd: standard deviation of baseline value for control group
#nc: number of participants included in the control group

metadat <- escalc (measure="SMD", m1i=trt_m, sd1i=trt_sd, n1i=nt,
m2i=con_m, sd2i=con_sd, n2i=nc, data=rcts)
rcts_b1_order<-rcts[order(metadat$yi),]
metadat1 <- escalc (measure="SMD", m1i=trt_m, sd1i=trt_sd, n1i=nt,
m2i=con_m, sd2i=con_sd, n2i=nc, data=rcts_b1_order)

rcts_b1_1<- metadat1[metadat1$study=="1",]
rcts_b1_2<- metadat1[metadat1$study=="2",]
rcts_b1_3<- metadat1[metadat1$study=="3",]
rcts_b1_4<- metadat1[metadat1$study=="4",]
rcts_b1_5<- metadat1[metadat1$study=="5",]
rcts_b1_6<- metadat1[metadat1$study=="6",]
rcts_b1_7<- metadat1[metadat1$study=="7",]
rcts_b1_8<- metadat1[metadat1$study=="8",]
rcts_b1_9<- metadat1[metadat1$study=="9",]
rcts_b1_10<- metadat1[metadat1$study=="10",]
rcts_b1_11<- metadat1[metadat1$study=="11",]
rcts_b1_12<- metadat1[metadat1$study=="12",]
rcts_b1_13<- metadat1[metadat1$study=="13",]
```

```
##### Guideline on Forest plot #####

metasum1 <- metacont (nt, trt_m, trt_sd, nc, con_m, con_sd,
data=rcts_b1_11, sm="SMD", comb.random = TRUE)

par (mar=c (0.001,0.001,0,0))
y<-seq (0.999,0.001, length = length(metasum1$TE) * 10000)
x<-qnorm(y2, mean=0, sd=metasum1$seTE.fixed)
plot (x,y, type="l", lwd= 4, col="red", xlim=c(-2,2), ylim=c(-0.1,1), yaxt="n", xlab="SMD", ylab="p",
axes=FALSE)

smd<-metasum1$TE; lci<- metasum1$lower; uci<- metasum1$upper
data2<-data.frame(smd, lci, uci)
data2_order<-data2[order(data2$smd),] #descending order
y1<-seq (1, length(metasum1$TE))
y2<-y1/(length(metasum1$TE) + 1)
points (0,1/2, col="red", pch=19, cex=1.5)
abline (v=0, col="black", lty=1)

weights<-(metasum1$w.fixed) * (100/sum (metasum1$w.fixed))
for (i in 1: length(metasum1$TE)) {
if (data2_order$smd[i] < 0) {points (data2_order$smd[i], y2[i], pch=15, cex=0.1*weights[i])}
else {points(data2_order$smd[i], y2[i], pch=0, cex=0.1*weights[i])}}
for (i in 1: length(metasum1$TE)) {
lines(c(data2_order$lci[i], data2_order$uci[i]), c(y2[i], y2[i]), lty=1, col="black")}

polygon (c (metasum1$lower.fixed, metasum1$TE.fixed, metasum1$upper.fixed,
metasum1$TE.fixed), c (-0.075, -0.09, -0.075, -0.06), col="black")
text (-1, 0.8, "# 11", pos = 2, cex=1.1, font=2)
abline (v=metasum1$TE.fixed, col="blue", lty=2)

##### Funnel plot exploration #####

metasum <- escalc (measure="SMD", m1i=trt_m, sd1i=trt_sd, n1i=nt,
m2i=con_m, sd2i=con_sd, n2i=nc, data=rcts_b1_8)
metares<-rma(yi,vi, method="FE", data=metasum)

par (mar=c (4,4,2,1))
funnel (metares, refline=0, cex.axis=0.8, cex.lab=0.8, xlab="SMD", ylab="SE", at=c(-1,0,1))
se <- seq (0, 1.8, length=100)
reg1<-regtest (metares, model="lm")
lines(coef(reg1$fit) [1] + coef(reg1$fit) [2] * se, se, lwd=2, col="red")
abline(v=metares$b, lty="dotted"); title("# 8",    cex.main=1)
```
